# The Evolution and Distribution of Microstructures in High-Energy Laser-Welded X100 Pipeline Steel

**DOI:** 10.3390/ma12111762

**Published:** 2019-05-30

**Authors:** Gang Wang, Limeng Yin, Zongxiang Yao, Jinzhao Wang, Shan Jiang, Zhongwen Zhang, Cunguo Zuo

**Affiliations:** 1 School of Metallurgy and Materials Engineering, Chongqing University of Science and Technology, Chongqing 401331, China; wwg_16@163.com (G.W.); yaozongx@163.com (Z.Y.); jinzhao_wang@foxmail.com (J.W.); JiangShan248@163.com (S.J.); hnzhangzhongwen@163.com (Z.Z.); zuodaguo@163.com (C.Z.); 2 Guangdong Provincial Key Laboratory of Advanced Welding Technology, Guangdong Welding Institute (China-Ukraine E.O. Paton Institute of Welding), Guangzhou 510650, China

**Keywords:** laser welding, X100 pipeline steel, CSL, grain orientation, recrystallization texture

## Abstract

High-energy beam welding was introduced for pipeline steel welding to reduce pipeline construction costs and improve the efficiency and safety of oil and gas transportation. Microstructures and their distribution in X100 laser-welded joints, which determine the joints’ strength and toughness, are discussed in this paper. Welded joints were prepared by an automatic 10,000-watt robot-based disc laser-welding platform for 12.8 mm thick X100 pipeline steel. Then, the grain, grain boundary, orientation, and distribution pattern of each zone of the welded joints were studied by optical microscopy (OM), scanning electron microscopy (SEM), energy-dispersive spectroscopy (EDS), and electron backscattered diffraction (EBSD) analysis techniques. The results showed that the grain boundary density, contents of the high-angle and low-angle grain boundaries, distribution states, and evolution trends of coincident site lattice (CSL) grain boundaries were essentially the same in each zone from the base metal (BM) to the weld of the X100 pipeline steel laser-welded joint. The relative content of grain boundaries above 55°, which were composed of the Σ3 type CSL grain boundary, showed a considerable impact on the mechanical properties of the joint. The content of twin grain boundaries was closely related to the thermal cycles of laser welding, and the effect of the cooling rate was greater than that of the process of austenization.

## 1. Introduction

Because of its efficiency and reliability, high-grade pipeline steel with a large diameter, large thickness, high strength, and high toughness is widely used for oil and gas transportation today [1,2]. At present, arc welding is mainly used in pipeline steel welding. Parts with a thick section have a heat sink effect, causing a high cooling rate. This leads to complex changes in the heat-affected zone (HAZ) of welds because of the limitations of the energy density and penetration capacity with arc welding and other conventional welding methods [3,4]; the HAZ undergoes grain coarsening and metallurgical changes while the weld pool regions rapidly solidify. These microstructural changes adversely affect in-service performance factors such as the reliability and durability of the welded joints of high-grade pipeline steel [5].

In laser welding, two adjacent or stacked metal pieces are fused together under an inert gas flow with or without addition of material to the weld line [6]. This has the advantages of fast welding speed, large depth, and small deformation. In recent years, laser welding has gradually come into use for pipeline steel welding, so it is extremely significant to study the effects of changing laser parameters on the microstructure and properties of welded joints. Currently, a number of experiments have been conducted to research the microstructure and properties of laser-welded joints of dissimilar metals such as Al/Ti [7]. Guarino et al. [8,9] analyzed the effectiveness of the laser treatment by fatigue testing, and established a numerical model to understand the relationship between the laser treatment parameters and the fatigue enhancement of the components. In addition, the welding operating cost can be reduced by approximately 43% with acceptable mechanical properties if the optimal welding conditions are used [10,11].

However, study on the microstructure of laser-welded joints of X100 pipeline steel has been rarely reported. The final microstructure will directly determine the toughness and service reliability of the laser-welded joints in high-grade pipeline steel [12,13,14], and study of the grain, grain boundary, strain, and their distribution patterns in each microstructural zone of a laser-welded joint in high-grade pipeline steel has become an important method to improve its toughness. Therefore, to ensure the safety of X100 pipeline steel in service, the evolution of microstructures and their distribution characteristics in X100 welded joints should be investigated.

In this paper, welded joints were prepared by an automatic 10,000-watt robot-based disc laser-welding platform for 12.8 mm thick X100 pipeline steel. Then, optical microscopy (OM), scanning electron microscopy (SEM), energy-dispersive spectroscopy (EDS), and electron backscattered diffraction (EBSD) analysis techniques were used for an in-depth exploration of the grain, grain boundary, strain, and their distribution patterns in each microstructural zone of the laser-welded joints in high-grade pipeline steel. This study will provide some good scientific guidance and references for control of the strength and toughness of high-grade pipeline steel under laser-welding conditions.

## 2. Materials and Methods

### 2.1. Characterisation of the Material

**Figure 1:**
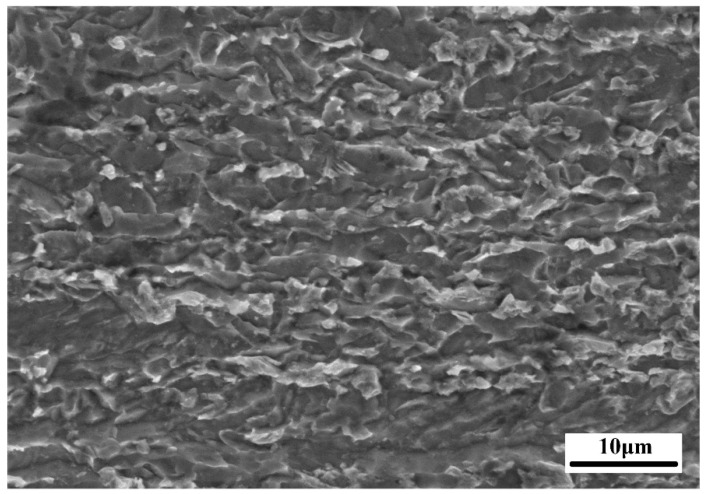
Microstructure of the X100 pipeline steel base metal.

The X100 pipeline steel meeting the API-5L standard was used, and its composition (wt%) is given in Table 1. Its production is based on the alloying technique and a thermo-mechanical control process (TMCP); the loss of strength due to the reduction in the carbon content was compensated for by adding trace amounts of alloying elements, and the comprehensive properties were improved by alloy phase-transformation strengthening, precipitation hardening, and fine-grain strengthening [15,16,17]. Its original microstructure was mainly dominated by bainite, including granular bainite, acicular ferrite, and a large number of martensite–austenite (M-A) constituents which were distributed between grains or inside the grains (Figure 1) [18,19].

### 2.2. Experimental Methods

**Figure 2:**
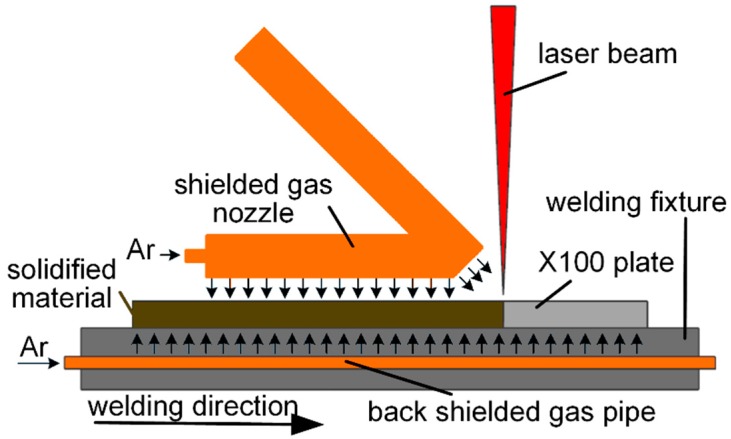
Schematic of the X100 laser-welding device.

The experiment was carried out using a double-sided laser-welding platform (TruDisk 10002, TRUMPF, Ditzingen, Germany, 10 kW, BEO D70 90° with a 200 mm collimator lens, a 200 mm focal lens, and a 200 μm transmission fibre. KR 60 HA high-precision si*x*-axis robot, KUKA, (Augsburg, Bavaria, Germany) was adopted by using the parameters shown in Table 2. The dimensions of the weld sample were 300 mm (RD) × 150 mm (TD) × 12.8 mm (ND), RD is the rolling direction, TD is the transverse direction, and ND is the normal direction. The samples were thoroughly cleaned before welding. As shown in Figure 2, the X100 plate is rigidly fixed by the welding fixture to avoid post-weld deformation and protected by a gas protection device during welding. The upper and lower surfaces are protected by pure argon supplied by shielded gas nozzle and back shielded gas pipe, respectively, while a drag mask is added to the upper surface to protect the welded surface. The back shielded gas pipe with holes is buried in the center of the welding fixture.

The welding was performed in a flat position (PA) on both sides. The laser power was 10 kilowatt (kW) in the front welding pass and 8 kW in the back welding pass. The joint was cut into metallographic samples with a size of 20 × 10 × 12.8 mm, followed by corrosion with 4% nitric acid. Subsequently, the sample was observed using OM (Axio Imager M2m, ZEISS, Oberkochen, Germany) and SEM (Nova NanoSEM 430, FEI, Hillsboro, OR, USA). EBSD (NordlysMax2, Oxford Instruments, Abindden, Oxford, UK) was used for the textural analysis of the microstructural zones (ion polishing for sample preparation, acceleration voltage 20 kV, scanning step size 0.5, and magnification 450), and MTEX software (Version 5.1.1, Free Software Foundation, Boston, MA, USA) was used to conduct the analysis and visualization of the EBSD measurement data [20].

## 3. Results and Discussion

### 3.1. Microstructural Zones of the Heat-Affected Zone (HAZ)

**Figure 3:**
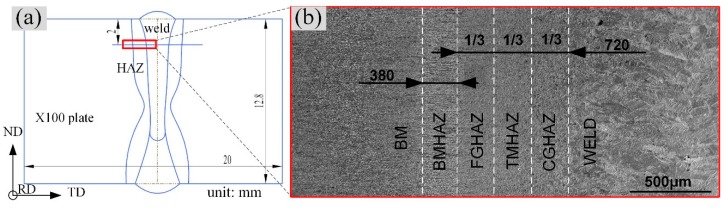
Components of the laser-welded joint in X100 pipeline steel: (**a**) shapes of the weld and heat-affected zone; (**b**) microstructural zones and sizes of the zones.

Under the action of a high-energy beam laser heat source, a significant difference and an apparent gradient in the microstructure were observed in the X100 pipeline steel from the base metal (BM) to the WELD in the joint. According to the microstructure images from OM, SEM, and EBSD and the welding thermal cycling process, the microstructure of the welded joints could be divided into six zones: the BM, the banded microstructure heat-affected zone (BMHAZ), the fine-grained heat-affected zone (FGHAZ), the transitional microstructure heat-affected zone (TMHAZ), the coarse-grained heat-affected zone (CGHAZ), and the WELD (Figure 3) [5,15,21]. Figure 3a shows a schematic diagram (equivalent ratio) of the cross-sectional profiles of the weld and HAZ in the X100 pipeline steel laser-welded joint. Figure 3b shows a magnified image of the feature area 2 mm away from the upper surface of the sample. At this location, the width of the HAZ was 1.1 mm, and the widths of the BMHAZ, FGHAZ, TMHAZ, CGHAZ, and WELD were 0.38 mm, 0.24 mm, 0.24 mm, 0.24 mm, and 1.6 mm, respectively.

The final microstructure that would determine the toughness of the joint depends on the austenization process of the original structure and the structural transformation process during continuous cooling after welding, and the effect of the extent of austenization is more significant than that of the cooling rate [16,17,22,23,24]. The heating rate, peak temperature, and high-temperature residence time of the weld gradually increase with decreasing distance from the centre or the upper surface of the weld, and the extent of austenization and cooling rate increase considerably; this leads the prior austenite grain size to become larger, but the size of the microstructure at room temperature becomes finer [19,23].

In the BMHAZ, incomplete austenization occurred, and a large number of M-A constituents were produced in the polycrystals (small grain size, long total grain boundary length, and high interface energy) at the locations with relatively high energy because it was close to the BM and away from the heat source centre (with good heat dissipation conditions, high temperature rising and cooling rates, and a low peak temperature). On the other hand, the relatively large ferrite grains at the locations with low energy continued to grow and retain the original microstructure, so the structures of the X100 pipeline steel in the BMHAZ were composed of ferrite and a banded M-A structure.

In the FGHAZ, austenization and recrystallization were completed. The microstructure was uniform and fine, the ferrite exhibited an equiaxed morphology, the granular M-A constituents were mainly concentrated at the grain boundary, and some M-A constituents were distributed in the ferrite grains. The austenite grains of the CGHAZ grew considerably, but fine lath-shaped bainitic ferrite (BF) in the prior austenite grain and granular M-A constituents dispersed between the grain boundary laths appeared during the rapid cooling process; a small number of quasi-polygonal ferrite (QF) blocks containing speckle M-A constituents also appeared. The prior austenite grain boundary was intact and distinct, showing a polygonal shape with a uniform grain size. The prior austenite grain boundary of the TMHAZ that was between the FGHAZ and CGHAZ was blurry and the grain size was different. Granular and massive ferrite and lath-like BF appeared, and granular M-A constituents appeared to be biased [5,15,21]. 

**Figure 4:**
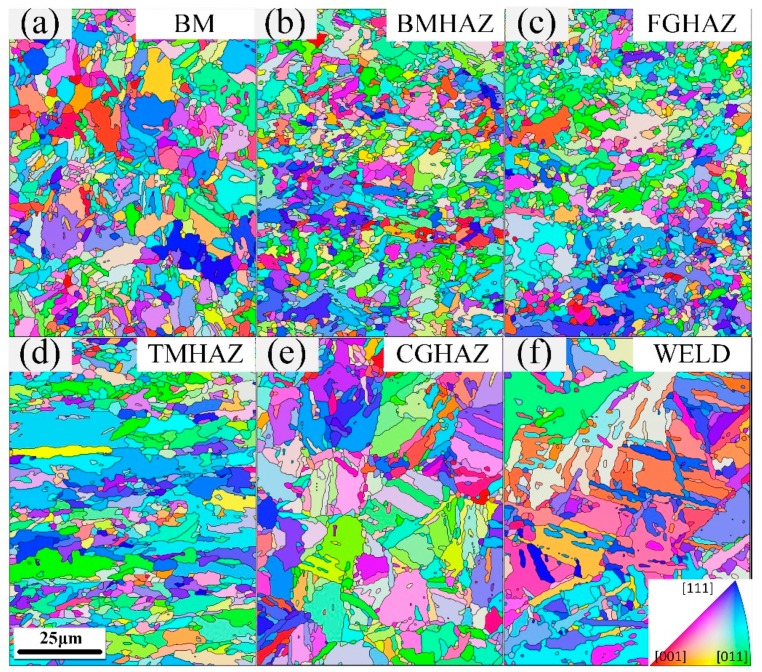
Grain orientation of each microstructural zone (from base metal (BM) to WELD) of the X100 laser-welded joint: (**a**) BM; (**b**) banded microstructure heat-affected zone (BMHAZ); (**c**) fine-grained heat-affected zone (FGHAZ); (**d**) transitional microstructure heat-affected zone (TMHAZ); (**e**) coarse-grained heat-affected zone (CGHAZ); (**f**) WELD.

As shown in Figure 4, significant differences in the grain size and orientation distribution between each microstructural zone could be found. As the distance to the weld decreased, the grain size increased gradually and the bulk and acicular microstructure appeared. Moreover, the EBSD test accuracy of the X100 laser welded joint was 0.2 μm. The grain boundary calculations were based on the 5° disorientation, and the data errors of the grain, grain boundary, and texture were less than 5%.

### 3.2. Size and Orientation of Grains

The microstructure of the X100 laser-welded joint contained ferrite and a small amount of retained austenite and martensite, and its strength and grain size were in accordance with the Hall–Petch relationship: $\sigma_{s}=\sigma_{i}+{\mathrm{kd}}^{-1/2}$, where $\sigma_{i}\;\mathrm{and}\;k$ are material-related constants and d is the grain size. Through this equation, the quantitative relationship between the microstructure and the material’s macroscopic performance could be determined. With decreasing equivalent grain radius, increasing grain boundary density, and increasing resistance to dislocation movement, the macroscopic strength and toughness are enhanced simultaneously [19,25,26].

**Figure 5:**
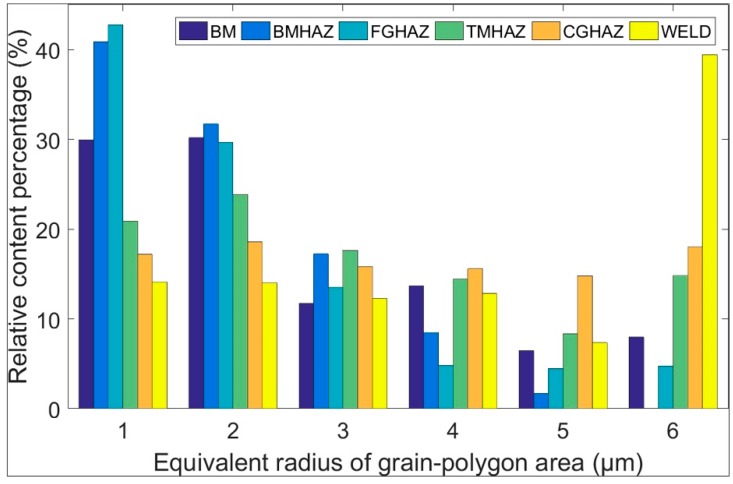
Histogram of the grain size distribution of each zone (from the BM to the WELD) of an X100 laser-welded joint.

A histogram of the grain size distribution of the X100 laser-welded joint from the BM to the WELD is shown in Figure 5. Grains with sizes of less than 2.5 μm, 2.5–4.5 μm, and greater than 4.5 μm accounted for 60.1%, 25.4%, and 14.5%, respectively, in the BM. The grain size distributions in the BMHAZ and FGHAZ were basically the same, and the content of grains smaller than 1.5 μm in the FGHAZ was approximately 2% higher than that in the BMHAZ. The contents of grains smaller than 1.5 μm in the BMHAZ and FGHAZ were 10.9% and 12.8% higher than that in the BM, respectively; the contents of grains with a size of 1.5 μm~2.5 μm in the BMHAZ and FGHAZ were basically the same as that in the BM; the contents of grains with a size of 2.5 μm~3.5 μm in the BMHAZ and FGHAZ were 1.8% and 5.5% higher than that in the BM, respectively; and the contents of grains with a size greater than 3.5 μm in the BMHAZ and FGHAZ were lower than that in the BM. The comprehensive properties of this area were excellent and its toughness was higher than that of the BM. From the TMHAZ to the WELD, the contents of grains smaller than 2.5 μm were much lower than that in the BM (15.4%, 24.3%, and 32.0%, respectively), and the contents decreased further with increasing grain size. The contents of grains larger than 2.5 μm from the TMHAZ to the WELD were higher than that in the BM, the content of grains larger than 5.5 μm in the WELD was 31.4% higher than that in the BM, and the toughness and strength of the area were lower than those of the BM. The grains in the CGHAZ were evenly distributed in size, and the content fluctuated from 11.7 to 17.3%. The lowest content of grains smaller than 2.5 μm was found in the WELD, and the content of grains with a size of 2.5–5.5 μm in the WELD was essentially the same as that in the BM. The content of grains larger than 5.5 μm in the WELD reached up to 39.4% (5 times that in the BM), and the maximum grain size was 12.73 μm.

### 3.3. Characterisation of the Grain Boundary

Grain boundaries are a type of surface defect in solid materials. According to the angle of the grain boundaries, they can be divided into low-angle grain boundaries and high-angle grain boundaries, and the subgrain boundary belongs to the low-angle grain boundaries. The high-angle grain boundaries can be further divided into coincident site lattice (CSL) grain boundaries and ordinary disordered grain boundaries according to the degree of atomic matching on the grain boundaries [15,16,27]. The influence of the grain boundary on the plastic deformation of the X100 pipeline steel laser-welded joint was reflected in the following: (1) the hindering effect of the grain boundary on sliding; (2) multiple slips caused by the grain boundary; (3) grain boundary sliding; (4) grain boundary migration; and (5) grain boundary segregation. Among these, the hindrance to sliding was the most significant. The macroscopically manifesting strength and hardness at the grain boundary were higher than those in the crystal because of hindrance to sliding leading to increased resistance to plastic deformation [28,29,30]. The microhardness of the X100 laser welded joint decreased first and then increased with the decrease of the distance from the weld center. The microhardness of the FGHAZ (average at 226 HV0.2) was lower than that of BM (about 233 HV0.2), while the average microhardness of the CGHAZ up to 302 HV0.2.

The grain boundary density in the BM of the laser-welded joint in X100 pipeline steel was 0.90 m/mm^2^, and the subgrain boundaries accounted for 5.39%. The grain boundary density in the BMHAZ was the highest, reaching 1.13 m/mm^2^, 25.56% higher than that in the BM, and the proportion of subgrain boundaries (accounting for 6.28%) was slightly higher than that in the BM. In the FGHAZ, the density was 1.04 m/mm^2^, 15.56% higher than that in the BM, and the proportion of subgrain boundaries (accounting for 5.54%) was basically the same as that in the BM. In the CGHAZ, the density was 0.74 m/mm^2^, 17.78% lower than that in the BM, and the subgrain boundary proportion was up to 10.52%, which was about twice as much as that in the BM. The grain boundary density and subgrain boundary proportion of the TMHAZ were between those of the FGHAZ and CGHAZ. The grain boundary density in the WELD was the lowest (0.67 m/mm^2^) among the zones, and the subgrain boundary proportion was slightly lower than that of the CGHAZ (9.12%).

**Figure 6:**
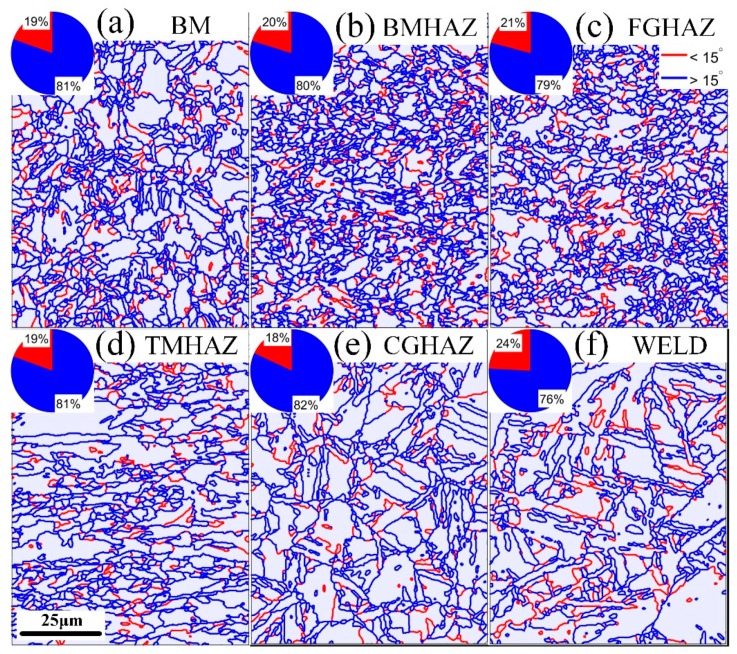
Distribution and proportion of high-angle and low-angle grain boundaries in BM (**a**), BMHAZ (**b**), FGHAZ (**c**), TMHAZ (**d**), CGHAZ (**e**), and WELD (**f**) of the X100 laser-welded joint.

The high-angle and low-angle grain boundary distributions and proportions of the X100 laser-welded joint from the BM to the WELD are shown in Figure 6. A grain boundary misorientation of 15° was used to divide the low-angle grain boundaries and high-angle grain boundaries, and the low-angle grain boundaries with a uniform distribution (specific distribution as follows: BM 19.94%, FGHAZ 20.17%, BMHAZ 20.87%, TMHAZ 19.40%, CGHAZ 18.02%, and WELD 24.16%) accounted for approximately 20%. The differences in the grain boundary density and grain size from the BM to the WELD were small, and the total grain boundary density near the FGHAZ was the largest.

In order to explore the influence of the microstructure on the toughness of X100 pipeline steel laser-welded joints, we further analysed the grain boundary orientation distribution, composition of the CSL boundaries, and twin boundaries as follows.

**Figure 7:**
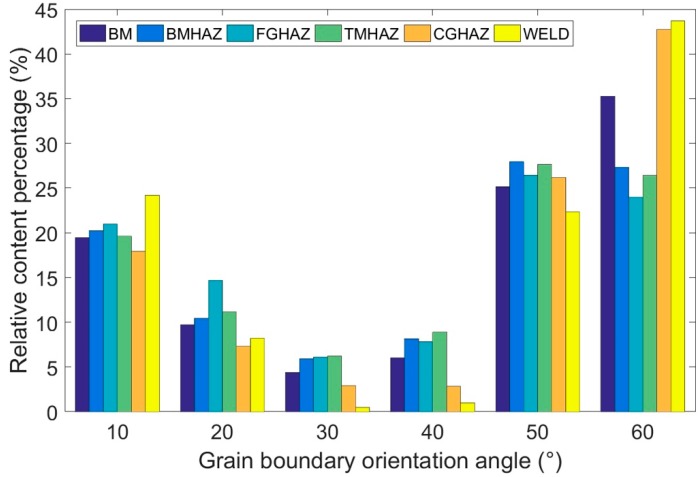
Distribution of the grain boundary misorientation in the X100 laser-welded joint from the BM to the WELD.

Figure 7 is a schematic diagram of the distribution of grain boundary misorientations from the BM to the WELD. The total distribution of high-angle grain boundaries with a misorientation greater than 15° was generally the same, but there were still differences between the different zones, especially for those boundaries with misorientations larger than 55°. In the BM, the grain boundary proportion first decreased and then increased as the misorientation angle increased, and the lowest point appeared near the misorientation of 30–40°; the proportion of grain boundaries greater than 55° was higher than that of the low-angle grain boundaries. From the BMHAZ to the TMHAZ, the distributions were basically the same when the grain boundary misorientation was 15–55°, and the corresponding grain boundary proportion was slightly higher than that of the BM, but the proportion was significantly lower than that of the BM (35.28%) for grain boundary misorientations greater than 55°. Among these zones, the proportion of corresponding grain boundaries in the FGHAZ at grain boundary misorientations of 15–25° was significantly higher than those in the other zones and reached approximately 15%. The distributions in the CGHAZ and WELD were essentially the same: When the grain boundary misorientation was between 15° and 55°, the corresponding grain boundary proportion was lower than those in the BMHAZ, FGHAZ, and TMHAZ. When the grain boundary misorientation was greater than 55°, the corresponding grain boundary proportion was higher than those from the BM to the TMHAZ. In particular, the proportion in the WELD was as high as 43.70%. According to the above analysis, the distributions of the grain boundaries with angles less than 55° were basically the same, but the distributions of the microstructures of the grain boundaries with angles larger than 55° were highly consistent with the mechanical properties; this indicates that the grain boundaries with angles larger than 55° exhibited the greatest impact on the toughness of the X100 pipeline steel.

A CSL grain boundary is a special interface, and a lattice satisfies the requirements of a CSL by sharing some lattice points. The CSL grain boundary is characterized by Σ, which is the ratio of CSL unit cells to standard unit cells. The value of Σ depends on the axis of the adjacent grains and the misorientation of the grain boundary. The frequency of occurrence of Σ grain boundaries is closely related to the crystallographic structure of a material, and control of the proportion and distribution of CSL grain boundaries in a material could significantly improve the material’s properties. The smaller the Σ value, the higher the frequency of occurrence. The more atoms there are at the grain boundary in the coincident lattice site, the denser the arrangement of grain boundary atoms; thus, there are fewer vacancies on the grain boundary and less adsorption of impurifying elements by the grain boundary, which improves the corrosion resistance and grain boundary strength [31,32,33]. 

**Figure 8:**
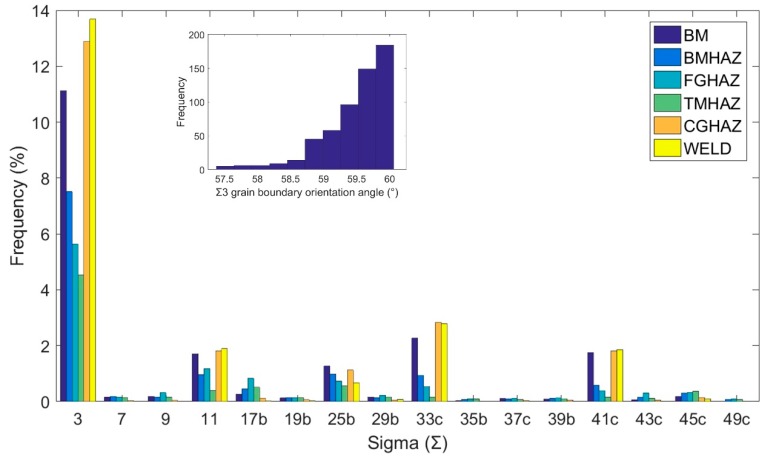
Histogram of coincident site lattice (CSL) distribution in each zone of the X100 laser-welded joint from the BM to the WELD.

According to the complete measurement results of the CSL grain boundaries in the high-angle grain boundaries of the X100 laser welded joints from the BM to the WELD, most of the CSL-type grain boundaries were exhibited at relatively low contents and only slightly affected the material properties. A distribution histogram of CSL grain boundaries with a relative content of more than 0.1% (total proportion of 92% or more) is shown in Figure 8. It can be seen that the relative contents of the different types of CSL in each zone of the X100 pipeline steel laser-welded joint showed basically the same trend. In the CSL grain boundaries with a high-angle grain boundary proportion of greater than 0.1%, Σ3, Σ11, Σ25b, Σ33c, and Σ43c were dominant, and their total proportion was above 70%. In particular, Σ3 accounted for more than 45% of the CSL grain boundaries. The grain boundary density of CSL in the BM was 0.14 m/mm^2^, which accounted for 20.10% of the high-angle grain boundaries. Among them, Σ3, Σ11, Σ25b, Σ33c, and Σ43c accounted for 90.18% and Σ3 accounted for 55.32% of the CSL grain boundaries. The misorientation of the Σ3 grain boundaries was between 57° and 60°, and the content of Σ3 increased sharply with increasing angle; the highest content of Σ3 grain boundaries was found near 60°. In the BM, the grain boundaries greater than 55° accounted for 35.28%; among them, Σ3 accounted for 11.12% (that is, 31.52% of the grain boundaries above 55°). The contents of CSL grain boundaries in the BMHAZ, FGHAZ, and TMHAZ decreased in descending order, and their values were 7.52%, 5.64%, and 4.53%, respectively; the lowest content was found in the TMHAZ and was approximately 40% of that in the BM. The relative contents of the CSL grain boundaries in the CGHAZ and WELD were significantly higher than that in the BM (12.90% and 13.69%, respectively), while their grain boundary densities (0.12 m/mm^2^ and 0.10 m/mm^2^, respectively) were still lower than that in the BM. In addition, as seen from Figure 7 and Figure 8, the histograms of the grain boundaries with a misorientation angle of greater than 55° in each zone were substantially identical to the content of Σ3, which indicated that Σ3 was an important component of the grain boundaries with misorientation angles above 55°.

The microstructure of the laser-welded joint in X100 pipeline steel was mainly composed of ferrite, and its twin system was {112}<$11\bar{1}$> [30,33]. The EBSD analysis results of X100 laser welded joints from the BM to the WELD are shown in Table 3. According to the data of the twin by EBSD, the density of the twin boundaries in the BM was 7.27 mm/mm^2^, accounting for 1.13%. In the BMHAZ, it was 15.46 mm/mm^2^, accounting for 1.91% and reaching 2.12 times as much as that in the BM. The twin boundary density in the FGHAZ (20.31 mm/mm^2^, accounting for 2.73%) was higher than that in the BMHAZ and was 2.79 times as much as that in the BM. In the TMHAZ, the density (13.22 mm/mm^2^) was slightly lower than that in the BMHAZ, but the proportion (2.28%) was increased. The proportions of twin boundaries in both the CGHAZ and WELD were 0.89%, and the twin boundary density in the CGHAZ was 4.49 mm/mm^2^. The lowest twin boundary density appeared in the WELD (3.82 mm/mm^2^)—about half of that in the BM. From the discussion above, we can see that the proportion of twin boundaries was closely related to the welding thermal cycles, and the impact of the cooling rate was greater than that of the extent of austenitization.

### 3.4. Recrystallization Texture

Texture is a state in which the grain orientation deviates considerably from a random distribution, resulting in anisotropy of the macroscopic properties. In order to obtain the texture type and distribution pattern of cubic crystal polycrystalline materials, the ODF (orientation distribution function) is used to measure and visualize the texture [34,35,36]. The texture index calculated by the ODF is an important parameter for describing the texture strength of polycrystalline metal materials [37,38,39].

**Figure 9:**
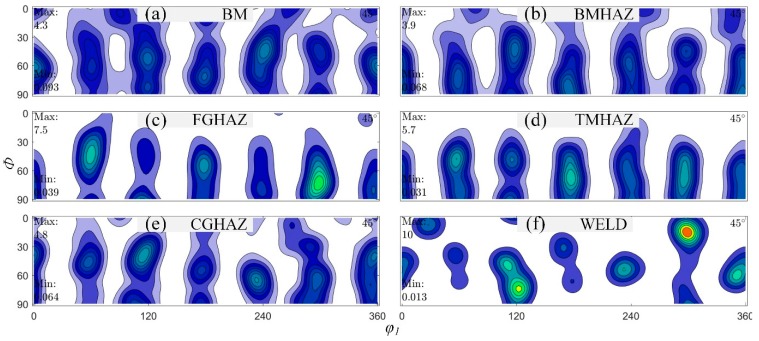
Orientation distribution function (ODF) cross section at φ_2_ = 45° of the X100 laser-welded joint from the BM to the WELD.

Figure 9 shows the ODF cross section at φ_2_ = 45° of the X100 laser-welded joint from the BM to the WELD. The BM was formed by rolling and had a texture index of 1.53 and a maximum texture strength of 4.28; there was a presence of a <110>//RD type fibre texture, and the proportions of the three texture components {$\bar{2}11$}<110>, {212}<$10\bar{1}$>, and {$\bar{1}02$}<$1\bar{1}0$> were 45.7%, 39.7%, and 14.6%, respectively. The maximum texture strength of the BMHAZ was 3.95, lower than that of the BM, but the texture index was increased to 1.61, indicating that the types and proportions of the texture had changed. The texture type gradually evolved from <110> type to <211> type and <210> type, and the proportion of <110> type textures decreased from 100% to 21.9%. The proportion of the single-transition-type fibre texture component <211> was the highest (34.1%), and the <211> type fibre texture consisted of {$\bar{1}21$}<210> and {$1\bar{2}2$}<101>, accounting for 26.7% and 17.3% (a total of 44.0%), respectively. The recrystallization process in the FGHAZ was complete, and the texture transformation was basically finished. The texture index reached 2.32, and the maximum texture strength reached 8.8, twice as much as that in the BM. The proportion of the <210> fibre texture was more than half (51.6%), the proportion of the <110> fibre texture was 25.4%, and the remaining 23.0% was the <211> texture. From the TMHAZ to the WELD, the texture composition was generally stable, essentially the same as in the FGHAZ. However, the proportions changed substantially. The proportion of the <210> type fibre texture increased from 31.6% to 54.5% and to 60.1%, and the proportion of the <110> type fibre texture decreased from 46.8% to 21.9% and to 19.5%. Due to the presence of WELD columnar crystals, the maximum texture strength was as high as 11.44, and the texture index also reached a maximum value of 3.04.

## 4. Conclusions

Control of the strength and toughness of laser-welded joints in high-grade pipeline steel is the key to the wide application of laser welding to high-grade pipeline steel welding. Meanwhile, the core of strength and toughness control lies in the microstructure and its distribution in the welded joint. By studying the microstructure and distribution characteristics of the X100 laser-welded joints, we came to the following conclusions:

(1) The grain sizes from the BM to the WELD exhibited a substantial difference. Grains larger than 5.5 μm in the WELD accounted for 39.4% of grains—5 times as much as that in the BM—and the maximum grain size was 12.73 μm.
(2) The distribution states and evolution trends of the grain boundary density, high-angle and low-angle grain boundaries, and CSL grain boundaries in the microstructural zones from the BM to the WELD were generally the same.
(3) The Σ3 type CSL grain boundary was an important component of the high-angle grain boundaries above 55°, and the relative content of grain boundaries above 55° showed the greatest influence on the mechanical properties.
(4) The relative content of twin boundaries in the X100 pipeline steel laser-welded joint was closely related to the welding thermal cycles, and the impact of the cooling rate was greater than that of the extent of austenitization.
(5) The main components of the pipeline steel laser-welded joint texture were the <210 >, <110>, and <211> fibre textures, and the <210> type fibre texture had the greatest influence on the mechanical properties.

In future work, we will focus on the mechanical properties of the X100 pipeline steel laser-welded joints in order to establish a quantitative relationship between the welding process, microstructures, and mechanical properties.

## Figures and Tables

**Table 1:** Chemical composition and carbon equivalent (CE) of X100 pipeline steel (wt%).

C	Mn	Cr	Si	Mo	Nb	Ti	V	Al	Ni	Cu
0.064	1.870	0.023	0.099	0.003	0.017	0.017	0.002	0.012	0.470	0.280
N	P	Co	Fe	CE ^a^	-	-	-	-	-	-
0.017	0.009	0.003	bal	43.13%	-	-	-	-	-	-

^a^ CE = C + Mn/6 + (Cr + V + Mo)/5 + (Cu + Ni)/15.

**Table 2:** Parameters of the X100 pipeline steel laser welding process.

Material (mm)	Laser Power (kW)	Welding Speed (m/min)	Defocusing Distance (mm)	Front Shielding Gas Flow (L/min)	Back Shielding Gas Flow (L/min)
X100 (12.8)	10	2.1	−4	15	25
	8				

**Table 3:** The results of grain boundary (GB) of X100 laser welded joints by using electron backscattered diffraction (EBSD).

	BM	BMHAZ	FGHAZ	TMHAZ	CGHAZ	WELD
GB density (m/mm^2^)	0.90	1.13	1.04	0.85	0.74	0.67
Sub GB content (%)	5.39	6.28	5.54	9.94	10.52	9.12
General GB content (%)	94.61	93.72	94.46	90.06	89.48	90.88
General GB density (m/mm^2^)	0.85	1.06	0.98	0.77	0.66	0.61
Low-angle GB content (%)	19.49	20.17	20.87	19.40	18.02	24.16
High-angle GB content (%)	80.51	79.83	79.13	80.60	81.98	75.84
High-angle GB density (m/mm^2^)	0.69	0.85	0.78	0.62	0.54	0.46
CSL GB content (%)	20.10	13.69	12.08	8.43	21.38	21.30
Σ3 GB content (%)	55.32	54.91	46.72	53.71	60.33	64.25
CSL GB density (m/mm^2^)	0.14	0.12	0.09	0.05	0.12	0.10
Twin GB density (mm/mm^2^)	6.60	14.07	14.23	11.43	4.04	1.36
Twin GB content (%)	1.02	1.74	1.91	1.97	0.80	0.32
Texture index	1.53	1.61	2.32	2.11	1.78	3.04
Maximum texture strength	4.28	3.95	8.80	5.91	5.08	11.44
